# Computed ABC Analysis for Rational Selection of Most Informative Variables in Multivariate Data

**DOI:** 10.1371/journal.pone.0129767

**Published:** 2015-06-10

**Authors:** Alfred Ultsch, Jörn Lötsch

**Affiliations:** 1 DataBionics Research Group, University of Marburg, Hans–Meerwein—Straße, 35032, Marburg, Germany; 2 Institute of Clinical Pharmacology, Goethe—University, Theodor—Stern—Kai 7, 60590, Frankfurt am Main, Germany; 3 Fraunhofer Institute for Molecular Biology and Applied Ecology IME, Project Group Translational Medicine and Pharmacology TMP, Theodor–Stern—Kai 7, 60596, Frankfurt am Main, Germany; Nankai University, CHINA

## Abstract

**Objective:**

Multivariate data sets often differ in several factors or derived statistical parameters, which have to be selected for a valid interpretation. Basing this selection on traditional statistical limits leads occasionally to the perception of losing information from a data set. This paper proposes a novel method for calculating precise limits for the selection of parameter sets.

**Methods:**

The algorithm is based on an ABC analysis and calculates these limits on the basis of the mathematical properties of the distribution of the analyzed items. The limits im-plement the aim of any ABC analysis, i.e., comparing the increase in yield to the required additional effort. In particular, the limit for set A, the “important few”, is optimized in a way that both, the effort and the yield for the other sets (B and C), are minimized and the additional gain is optimized.

**Results:**

As a typical example from biomedical research, the feasibility of the ABC analysis as an objective replacement for classical subjective limits to select highly relevant variance components of pain thresholds is presented. The proposed method improved the biological inter-pretation of the results and increased the fraction of valid information that was obtained from the experimental data.

**Conclusions:**

The method is applicable to many further biomedical problems in-cluding the creation of diagnostic complex biomarkers or short screening tests from comprehensive test batteries. Thus, the ABC analysis can be proposed as a mathematically valid replacement for traditional limits to maximize the information obtained from multivariate research data.

## Introduction

A recurring problem in biomedical research is the high dimensionality of data sets and the complexity of derived results. Multivariate data sets often differ in several factors or derived statistical parameters, which have to be selected for a valid interpretation. This selection is usually based on contextual and mainly traditional statistical limits. This leads occasionally to the perception of losing information from a data set; however, crossing the accepted statistical limits will be rejected almost certainly by a scientific audience. Dealing with the problem of statistical limits is an active research topic; however, the correct statistical approach at a rational selection of the most informative set of variables derived from multivariate analyses is not obvious. Scientists are therefore often inclined to use conservative statistical selection criteria to avoid α error. This is widely accepted but has a tendency toward occasionally disregard of valid information from experimental data.

Therefore, a theoretical basis for the selection of parameter sets that are interpretable in multivariate data is highly desirable to identify the optimum information that can be validly retrieved from biomedical data. The present report proposes a novel method that uses concepts developed in economical sciences. In particular concepts are used in the search for a minimum possible effort that gives the maximum yield. In many circumstances it has been observed that this converges toward the effect that with 20% of the effort 80% of all yield can be obtained, which is commonly called the “Pareto 80/20 rule” [1,2]. A more general approach is the so-called “ABC analysis”, which divides the data set into the three disjoint sets A, B and C, in such way that set “A” should contain the “important few”while set “C” contains the “trivial many” [2].

The determination of the set limits for an ABC analysis has so far been left to subjective considerations. In this paper, a calculation method is presented that allows calculating these limits on the basis of the mathematical properties of the distribution of the analyzed items. The utility of the proposed method will be illustrated by an example from own previous research [3] where this method improves the biological interpretation of the results and increased the fraction of valid information that can be obtained from experimental data. Further biomedical applications, such as deriving screening tests from complex test batteries, will be discussed.

## Methods

### Properties of ABC curves

**Fig 1:**
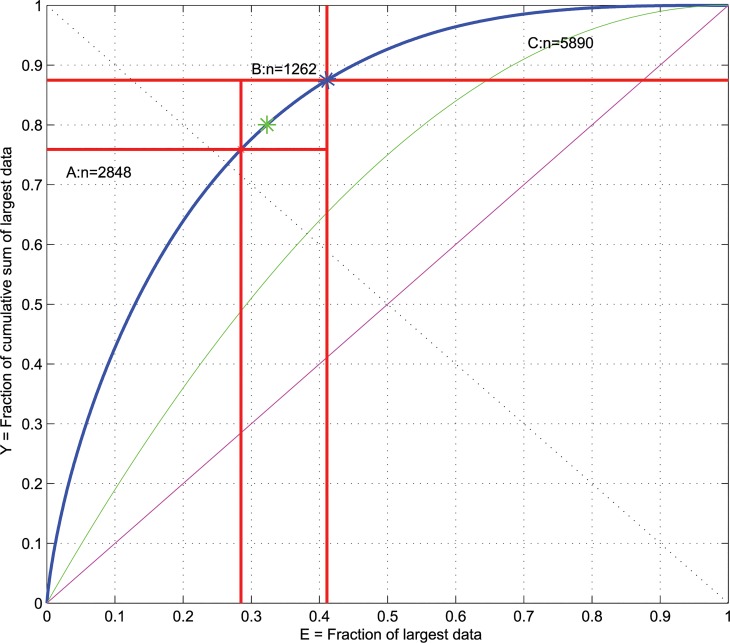
ABC plot of n = 10,000 data points, *x_i_*, drawn from the $\chi_{1}^{2}$ distribution (see also Fig 3). In addition, APC plots of the identity distribution, *x*_*i*_ = constant (magenta line), and of the uniform distribution in the data range *U[min(xi)*,*max(xi)]* (green line) are shown. The second main diagonal (dashed line) delivers the points where the yield *Y* equals the remaining effort described as *Y = 1—E*. The *BreakEven* point, i.e., the point on the ABC curve where the profit gain *dABC* equals 1 which means that the slope of the ABC curve at this point equals a value of 1, is marked a as green star. The limits of sets A, B and C resulting from the present ABC analysis are drawn as red lines.

The selection of the most prominent components of a PCA is a special case of a common problem met during multivariate data analysis. Let *x*_*1*_,…, *x*_*n*_ be a set of n positive values (*x*_*i*_ > 0) that describe n different variables of an empirical data set with respect to properties such as “importance”, “weight”, “effect” or “yield”. The distribution of the values *x*_*i*_ is unequal, i.e., few *x*_*i*_ have very large values while many *x*_*i*_ have small values. This can be plotted by means of ABC curves where x_i_ are sorted in decreasing order, *x*_*i*_ ≥ *x*_*i*+ 1_.The fraction of the first *i* elements to *n*, *E*_*i*_ = *i/n*, represents costs or “efforts”, *E*_*i*_, while the fraction of the cumulative sum of the *x*_*i*_, relative to the total sum, is called the “yield”, *Y*_*i*_, of *x*_*1*_,*…x*_*i*_ obtained as $Y_{i}=\frac{\sum_{k=1}^{i}xi}{\sum_{i=1}^{n}xi}$. An ABC curve [4] is a plot of *Y*_*i*_ versus *E*_*i*_ (Fig 1) as a special form of a graphical representation of cumulative distributions [5,6].

ABC curves are always non-decreasing concave functions in the unit square space. They are scale-invariant in the values of *x*. That is, a multiplicative modification of the x-values does not modify the ABC curve, i.e., *ABC(x) = ABC(x∙c)* for any c ≠ 0. However they are not invariant to the location of *x*. That is, an additive modification of the values of *x* does modify the ABC curve, i.e., *ABC(p)* ≠ *ABC(p+c)* for any c ≠ 0. For *x*_*i*_ *’ = x*_*i*_ *+ c* with c > 0, the ABC curve *ABC(x’)* will flatten and approach the identity distribution (green line in Fig 1). A special case is observed when x_i_ approaches a value of 0. Then, the curve approaches a “winner-takes-it all” distribution, i.e., tends to take a path through the points (0,0)—(0,1)—(1,1). Thus, the localization of ABC curves of a given data set relatively to (i) the identity distribution, i.e., all *x*_*i*_ have the same value *x*_*i*_ = *c*, and (ii) the uniform distribution, i.e., all values that *x*_*i*_ can take are equally likely in the interval from *min(x*_*i*_ *)* to *max(x*_*i*_ *)*, can be used to investigate the inequality of a distribution (Fig 1). Specifically, for all more right-skewed data distributions than the uniform distribution, the ABC curves will be located more toward the upper left corner of the plot.

To further describe ABC curves, their relation to Lorenz curves can be used [7]. For a probability density function *pdf(x)* and the cumulative distribution function *cdf(x)* with a (generalized) inverse *icdf(F)* the Lorenz curve *L(cdf(x))*, respectively *L(F)* is given as

$$\text{L}\left(\text{cdf}\left(\text{x}\right)\right)=\frac{\int_{-\infty }^{\text{x}}\text{t}\;\text{pdf}\left(\text{t}\right)\text{dt}}{\int t\;pdf\left(t\right)dt},\;\text{respectively}:\;\text{L}\left(\text{F}\right)=\frac{\int_{0}^{\text{F}}\text{icdf}\left(\text{F}\right)\text{dF}}{\int_{0}^{1}\text{icdf}\left(\text{F}\right)\text{dF}}.$$

ABC curves are related to Lorenz curves as follows: Let *L(p)* be a Lorenz curve for a probability distribution. The corresponding ABC curve *ABC(p)* can be derived as *ABC(p) = 1—L(1—p)* and vice versa *L(p) = 1—ABC(1—p)*. Following this interrelations, theoretical properties of ABC curves can be derived from the corresponding Lorenz curves. Moreover, analytical derivations of well-known distributions available for Lorenz curves can also be used for ABC curves (Table 1).

**Table 1:** ABC curves, *ABC(p)*, for some common distributions and their corresponding cumulative distribution functions, *cdf(x)*, as well as Lorenz curves, *L(p)*.

Distribution	cdf(x)	ABC(p)	L(p)
Equality	$\left\{ \begin{array}{cc} 0 & x\leq c\\ 1 & x>c \end{array}\right.$	p	p
Exponential	1 − *e* ^*λ*∙*x*^	*p* − *p* ∙ *ln*(*p*)	*p* + (1 − *p*) ∙ *ln*(1 − *p*)
Pareto	$1-{\left(\frac{x_{min}}{x}\right)}^{\alpha }$	$p^{\frac{\alpha -1}{\alpha }}$	$1-{\left(1-p\right)}^{\frac{\alpha -1}{\alpha }}$
Uniform in [a..a+b]	$\frac{x-a}{b}$	$\frac{0.5\;bp^{2}+(a+b)p}{a+0.5b}$	$\frac{0.5\;bp^{2}+ap}{a+0.5b}$
Uniform in [0..b]	$\frac{x}{b}$	−*p* ^2^ + 2*p*	*p* ^2^

### Calculation of precise limits for ABC analysis

An ABC analysis aims at identifying the minimum possible effort that gives the maximum yield. It divides the values *x*_*1*_, …, *x*_*n*_ into three disjoined sets A, B, and C [8]. Set A should contain the “critical few”, i.e., those elements that allow obtaining a maximum of yield with a minimal effort [1,2]. Set B comprises those elements where an increase in effort is proportional to the increase in yield. In contrast, set C contains the “trivial many”, i.e., those elements with which the yield can only be achieved with an over-proportionally large additional effort. The determination of these sets has been so far left to subjective judgments [8,9].

The derivation of statistically justified set limits regards the increase in “yield”(*Y*) versus the increase in “effort” (*E*). Formally, this is the first derivative (slope) of the ABC curve (*dY/dE = dABC*), in the following called “profit gain”. Set A should contain profit gains > 1 (*COND1*), set B should contain profit gains around a value of 1 (*COND2*), while the profit gain in set C should be substantially less than 1 (*COND3*). During ABC analysis the yield *Y* should be maximized while the necessary effort *E* minimized. Thus, to obtain the limit between sets A and B two variables need to be optimized. Moreover, as maximizing *Y* can be achieved via maximizing the unrealized yield *UY = 1 –E*, the optimization problem can be reduced to concomitantly minimizing both, *E* and *UY*.

#### Derivation of the limit between sets A and B

**Fig 2:**
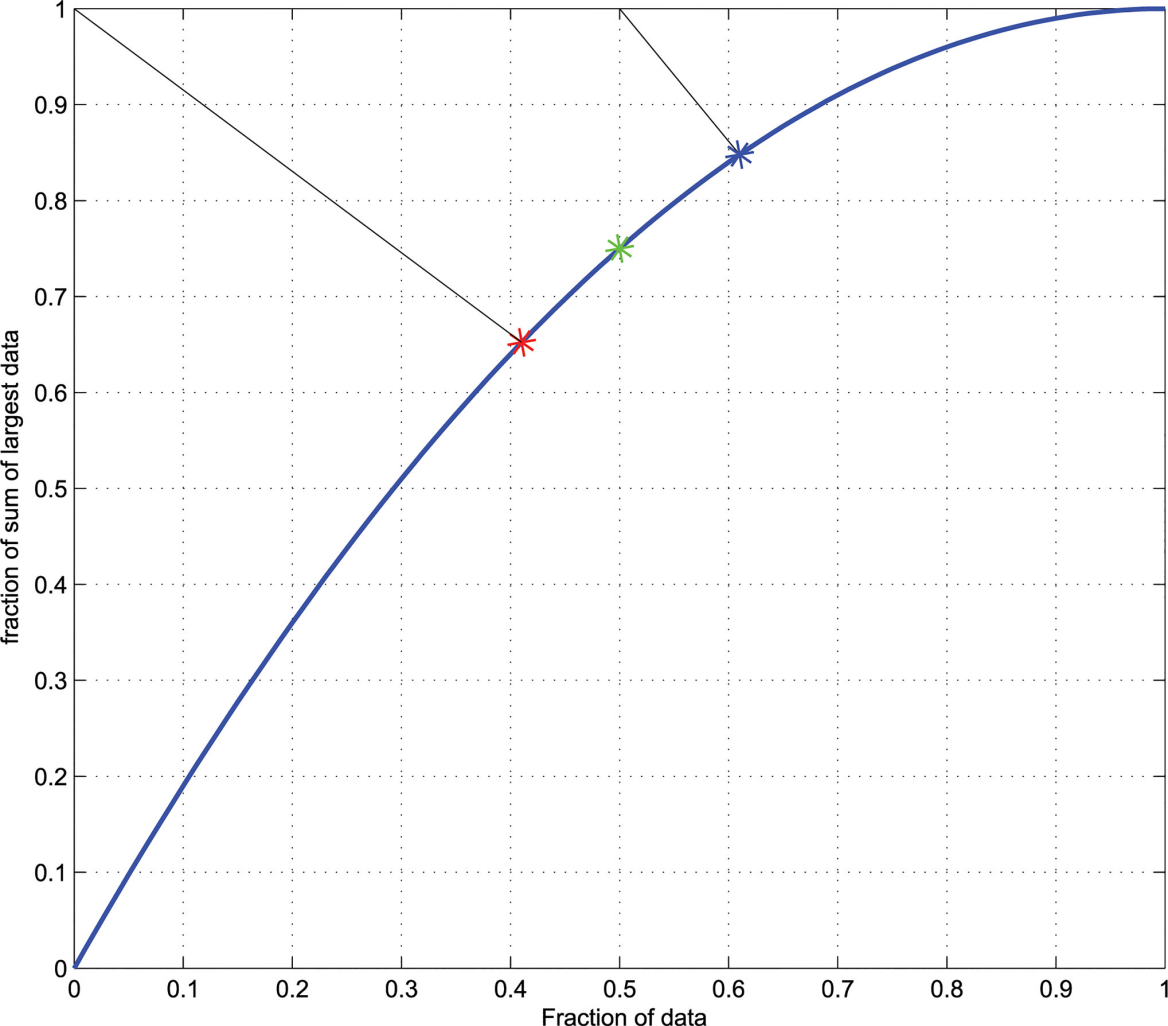
The ABC curve for *x*_*i*_ drawn from an uniform distribution *U[0*,*m]*, i.e., the drawn values are equally likely in the range from 0 to *m* with *x*_*1*_, …, x_*n*_ being a set of n positive data values (*x*_*i*_ > 0) sorted such that *x*_*i*_ ≥ x_*i+ 1*_. The fraction of the first *i* elements to *n (i/n)* represents costs or efforts (*E*_***i***_ = *i/n*), the fraction of the cumulative sum of the first xi values with regard to the total sum of the x_***i***_ is called the yield, *Y*_***i***_, of the set *x*_***1***_,*…x*_***i***_. The ABC curve (blue line) is a plot of *Y*_***i***_ versus *E*_***i***_. Intermediate points are interpolated by means of quadratic splines [7]. The ABC curve is independent of m. The red star marks the so called Pareto point *A(A*_***x***_,*A*_***y***_ *)*, i.e., the point at the smallest distance (left oblique black line) to the ideal point at, xy, *E = 0* and *Y = 1*. The green star marks the point on the ABC curve where its slope, *dY/dE*, equals 1. At this point, the profit gain *dABC* equals 1, therefore it is called the *BreakEven* point *B(Bx*,*By)*. The blue star marks the point *C(C*_***x***_,*C*_***y***_ *)* that has the smallest distance to the ideal situation where all gain has been achieved, i.e., E = B_***x***_ and Y = 1 (right oblique black line).

The derivation of the A/B set limits will be elaborated at the continuous uniform distribution *U = Uniform [0*,*m]* where the data points *x* are drawn with uniform probability $\frac{1}{m}$ within the interval [0,m] (Fig 2). The ABC curve of *U* is given by *ABC*(*p*) = −*p* ^2^ + 2*p* (Table 1). Note that this curve is independent of the limit *m*. The profit gain of this distribution is *dABC*(*p*) = −2*p* + 2, i.e., it starts at 2 (p = 0) and decreases to zero (p = 1) with a gradient of -2. An ideal limit for an ABC analysis is the point with zero effort (*E = 0*) and maximum effect (*Y = 1*), i.e., *ABCideal = (0*,*1)*. Hence, the optimization problem can be formulated as a distance point of the ABC curve to the *ABCideal* point. There are two immediate possible choices of a suitable distance function, namely (i) the Manhattan distance [10] consisting of the sum of the differences in *x* (*∆x*) and *y* directions (*∆y*; i.e., *∆x +∆y*) and (ii) the Euclidean distance represented by $\sqrt{{∆x}^{2}+{∆y}^{2}}$. The Manhattan distance leads to the optimization of *distM* = *E + (1-ABC)* whereas the Euclidean distance leads to the optimization of $distE=\sqrt{E^{2}+{(1-ABC)\;}^{2}}$. The minimization of either distance functions provides possible choices for the limit of set A. However, the minimum of *distM* leads to a profit gain limit of exactly 1, which fails to fulfill the condition for a valid definition of set A for which the profit gain should be greater than 1 (*COND1*). In contrast, minimization of *distE* results in profit gain of 1.18, which meets the above requirements and was therefore selected.

The point on the ABC curve which has the smallest (Euclidean) distance to *ABCideal* is called the Pareto point *A(A*_*x*_,*A*_*y*_ *)*. Its x-value, *A*_*x*_, provides a precise limit for data points in the set A. The point on the ABC curve where the profit gain *dABC* equals a value of 1 is called the “*BreakEven”* point *B(B*_*x*_,*B*_*y*_ *)*. Usually *A*_*x*_ *≤ B*_*x*_ holds in practice. However, this cannot be guaranteed for all possible distributions. In the case of *A*_*x*_ *> B*_*x*_ the points A and B exchange their role in ABC analysis. This procedure assures *COND1* for all distributions, i.e., for *x*_*i*_ *≤ A*_*x*_ the “profit gain” is ≥ 1.

From this derivations the set limit between sets A and B, *t*_*AB*_, is given as *min(A*_*x*_, *B*_*x*_ *)*. Set A is defined as *A = {x*_*i*_ *| x*_*i*_ *≤ icdf(X*, *t*_*AB*_ *100)} where *icdf(X*,*p)* for X = {*x*_*1*_, …, *x*_*n*._ *}* is the x_i_ corresponding to the p^th^ percentile. Set A contains the largest values of x_i_ down to a point where the ABC curve is closest to the ideal situation of zero effort and complete yield, as long as the *ProfitGain* is larger than 1. For the Uniform distribution set A contains the largest 41% of all values (Fig 2, red star). A geometric interpretation of the set limit between A and B is the point on the ABC curve that has the smallest distance from the ideal point (0,1; black line to the red star in Fig 2).

#### Derivation of the limit between sets B and C

According to the characteristics of an ABC analysis, the profit gain in set C should be substantially less than 1 (*COND3*). At an ideal point *B*_*y*_ = *1*, called the *BreakEven* point, all yield would be gained. The point on the ABC curve at a minimum distance from this ideal point (*B*_*x*_,*1*) is called *SubMarginal* point (C_x_ ,C_y_). For all points to the right of *C*_*x*_, i.e., *x*_*i*_ *≥ C*_*x*_, the profit gain is substantially less than 1 as required by *COND3*. From this derivations the set limit between sets B and C is given by *t*_*BC*_ = *C*_*x*_ and set C is *C = {x*_*i*_ *| x*_*i*_ *> icdf(X*, *t*_*BC*_ *100))}. For the Uniform distribution, set C contains the smallest 38% of the values (Fig 2 blue star). The remaining values of *x*_*i*_, neither associated to set A nor to set C, have to belong to set B. This set contains the values “around” a profit gain of 1 (*COND2*). For the uniform distribution these profit gains are in the range of 0.78 to 1.18. A geometric interpretation of the set limit between B and C is determined by the point at the smallest distance to the ideal point (*B*_*x*_,*1*; black line to the blue star in Fig 2).

## Results

The programs used to calculate the following ABC curves, which also perform the described precise ABC analysis and draw ABC plots, are part of the R package “ABCanalysis” (M. Thrun, Marburg, Germany) published on CRAN at http://cran.r-project.org/web/packages/ABCanalysis/index.html.

### ABC analyses of known distributions

**Fig 3:**
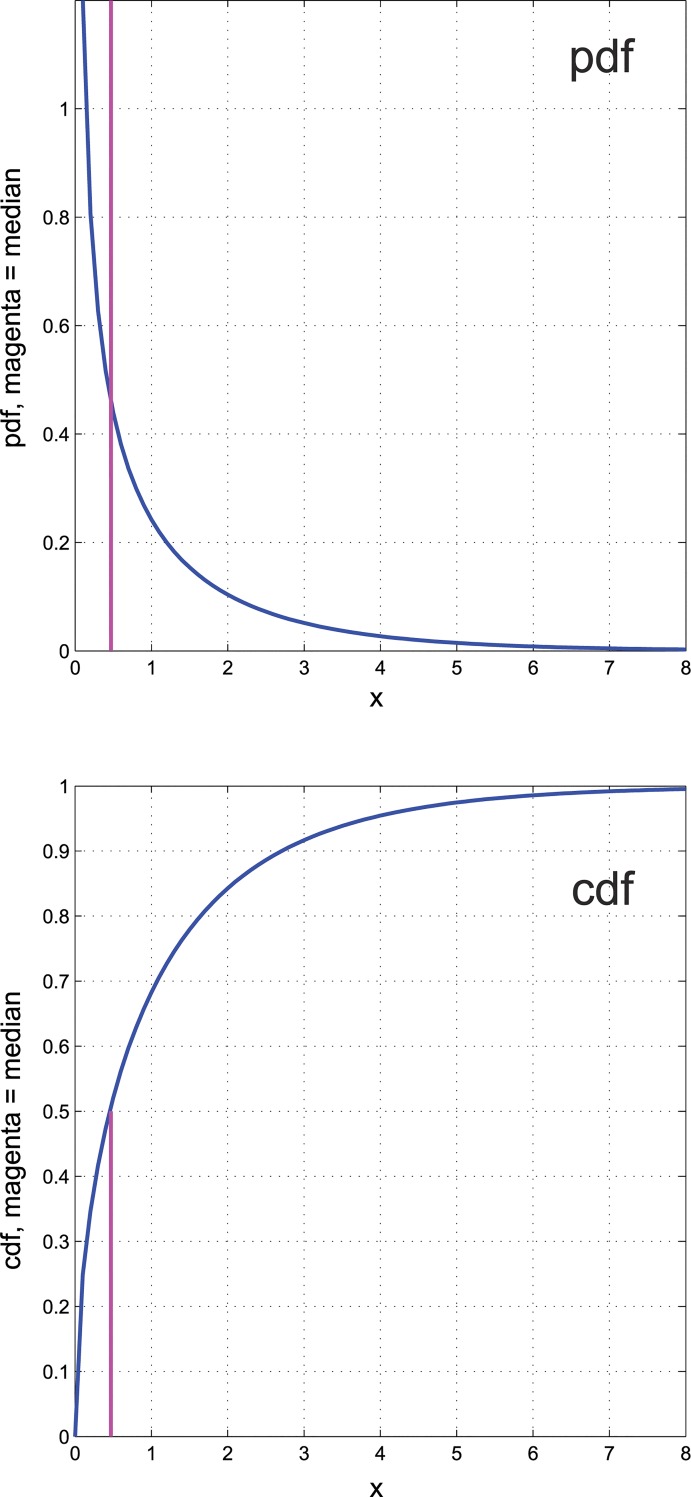
The probability density function (*pdf*) of the Chi square ($\chi \frac{2}{1}$) distribution (top panel) and its cumulative density function (*cdf*, bottom panel). This probability distribution is a typical example of a right skewed inequality distribution. The magenta lines indicate the location of the median (0.47) of the distribution (see also Fig 1 for comparison with less skewed distributions).

A commonly met distribution of data or derived statistical parameters is the chi-squared distribution with one degree of freedom, $\chi_{1}^{2}$ (Fig 3). This distribution is unequal to a large extent. Its median takes a value of 0.47, which means that 50% of randomly drawn data from this distribution are below 0.47 while the remaining 50% are right skewed distributed within the range of 0.47 and 8. Thus, half of the data concentrate within approximately 20% of the range across the other half of the data is distributed. Relative to the ABC curve of uniform distributions, the ABC curve of the $\chi_{1}^{2}$ distribution is located toward the upper left corner of the plot (Fig 1), which clearly shows that it is more right-skewed than the uniform distribution. For the $\chi_{1}^{2}$ distribution the *BreakEven* point is at approximately 32%. In set A, which contains approximately 24% of the data, the profit gain is more than 115%. In set C that contains more than 44% of the data the profit gain is less than 68%.

**Fig 4:**
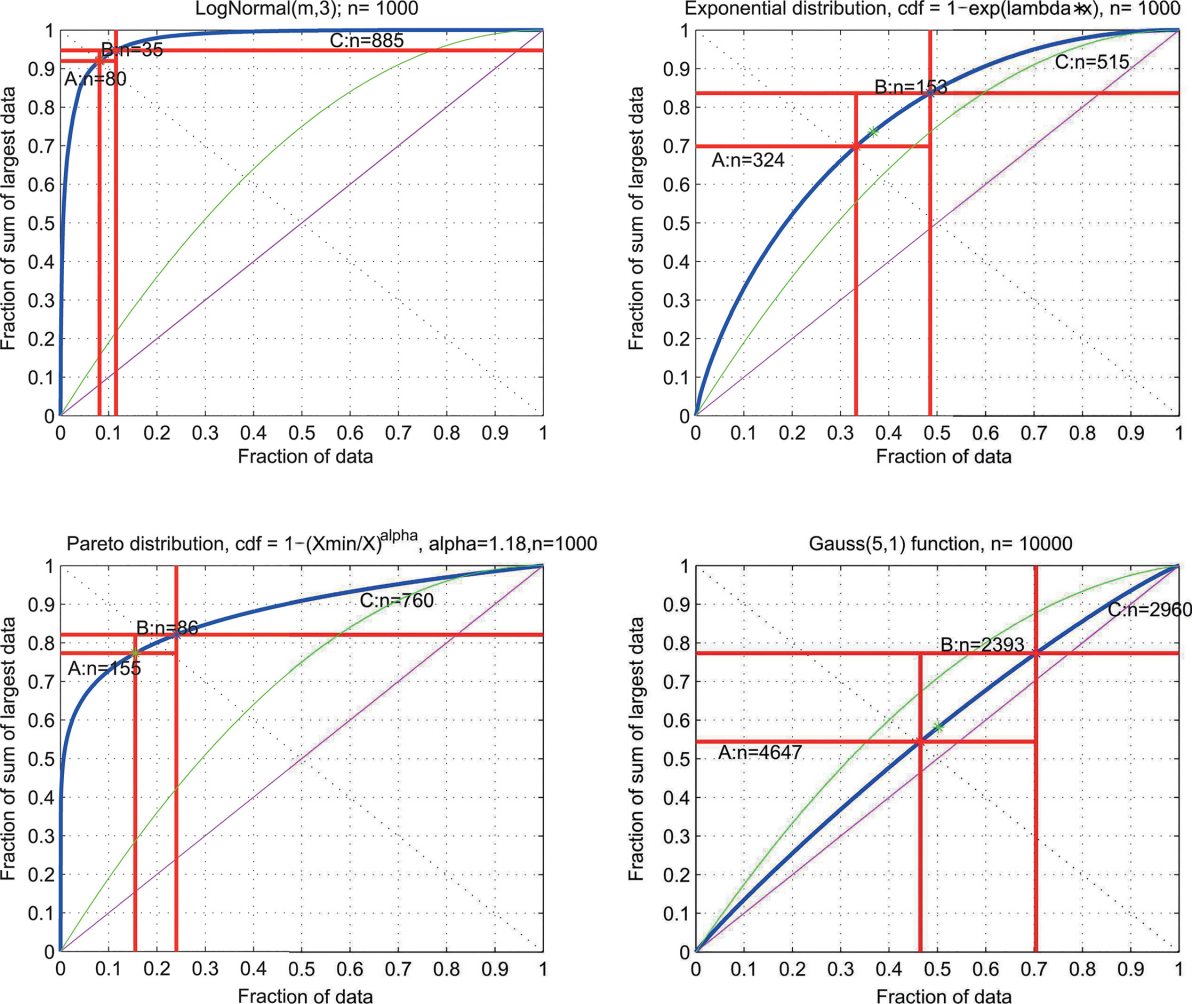
ABC plots for selected common distributions. For comparison, the ABC plots for the uniform distribution, *U = Uniform [0*,*m]*, where the data points *x* are drawn with uniform probability $\frac{1}{m}$ within the interval from 0 to a maximum of *m*, and for the identity distribution i.e., all *x*_***i***_ have the same value *x*_***i***_ = *c*, are shown as green and magenta lines, respectively. **Left upper panel:** ABC plot of the Lognormal distribution *LN(m*,*s)* with s = 3. The ABC curve for *LN(m*,*s)* is independent of *m*. **Upper right panel:** ABC plot of the family of exponential distributions with *cdf* = 1 – *e* ^*λx*^. The ABC curve for these distributions depends on the value of λ. **Lower left panel:** ABC plot of a Pareto distribution with $=1-{\left(\frac{x_{min}}{x}\right)}^{\alpha }$. For α = 1.18 the ABC curve passes through the “Pareto” point (see last paragraph of the discussion) at 20/80%). **Lower right panel:** ABC plot of a Gaussian distribution *N(5*,*1)*. This distribution shows lower inequality than the uniform distribution (green line) and comes close to the identity distribution (magenta line).

Further standard distributions can also be described with the present analysis (Fig 4). Firstly, among the distributions with most inequality is the *LogNormal* distribution family *LN(m*,*s)*. For *LogNormal* distributions the ABC curves depends only on the scale parameter *s*. As an example, Fig 4 shows in the upper left part the ABC plot for *LN(m*,*3)*. Less than 8% of the data belong to set A while approximately 90% belong to set C. If *s* is increased, the ABC curve passes very close to the *ABCideal* point of (0,1). Secondly, the ABC curve of the family of exponential distributions with a cumulative distribution function *cdf* = 1 – *e* ^*λx*^ has the form *ABC(p) = p–ln(p)* (Table 1) and is independent from λ. It shows less inequality than *LN(m*,*3)* (upper right panel of Fig 4), however, it is more unequal than the uniform distribution. Thirdly, for the Pareto distribution family with a cumulative distribution function $cdf=1-{\left(\frac{x_{min}}{x}\right)}^{\alpha }$, the form parameter α can be adjusted such that the ABC curve passes through the Pareto, or better Juran point ([2], see discussion), of effort = 20% and yield = 80% (lower left panel of Fig 4). Set B encompasses this point. Finally, Gaussians *N(m*,*s)* are among the most frequently used distributions. However, these distributions are not inequality distributions. In particular, if *s* is small as compared to *m*, then the data drawn from such distributions will resemble more an identical distribution with *c = m* and a few small deviations. In ABC plots, this is reflected by an inequality between the uniform distribution (Fig 4, lower right panel, green line) and the identity distribution (Fig 4, lower right panel, magenta line). For example, the *BreakEven* point for a Gaussian distribution of *N(5*,*1)* is located at *B*_*x*_ = 50% and with 47% of the data set A is larger than in other example distributions.

### ABC analysis of biomedical sample data

**Fig 5:**
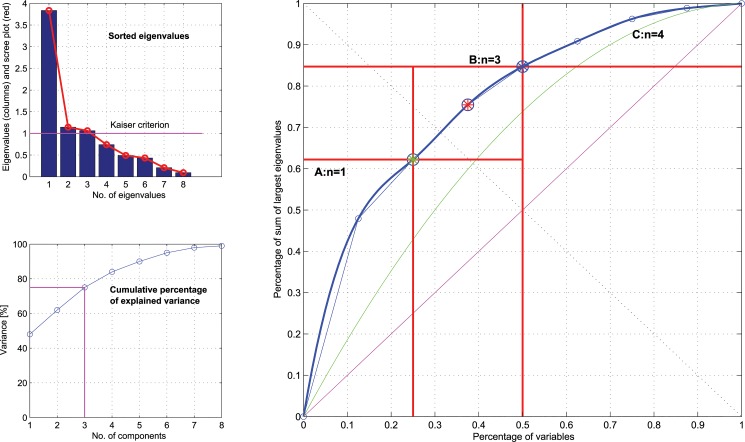
ABC analysis for a rationale selection of components for principal components analysis. **Left panel:** sample data showing the original results of a principal component analysis (PCA) of the covariance matrix among six different measurements of pain thresholds [3]. For classical selection of the set of relevant (largest) eigenvalues, either the Kaiser-Guttman criterion (eigenvalue > 1, **left top panel**, magenta line) [11,12] or the “elbow criterion” [13] in a so-called scree plot of the absolute values of eigenvalues sorted by descending size (left top panel, red curve) can be used. According to the PCA results, the sources of variance of pain thresholds to different nociceptive stimuli comprise eight variance components (see Table 2 in [3]). The **left bottom panel** shows the *cumulative* fraction of the variance explained by the principal components, with indication of the variance explained by the components that could be selected based on the classical criteria. It can also be seen that the inclusion of a further principal component would have provided a better coverage of the total variance. **Right panel:** ABC plot of the same data. The ABC curve (blue line) shows from bottom to top the increasing faction of the total sum of all eigenvalues and from left to right the increasing fraction of the total number of variance components which contribute these eigenvalues. The goal of the analysis is to identify the number of variance components (abscissa) that is associated with a satisfactorily high coverage (ordinate) of the total sum of available eigenvalues. Set A contains the largest data points, corresponding to the largest proportion of yield. For this particular distribution the set limit for A is obtained by the point with slope of the ABC curve of 1 (green star) resulting in the two largest eigenvalues. The set B consist of the next two eigenvalues including the Pareto point, i.e., the point closest to (0,1) (red star). In the present example the cumulative variance of component #1, 2and 3 contributed 75% to the total variance. The inclusion of component #4 results in a cumulative explained variance of 84%. On the other end, set C contains 50% of the eigenvalues, which share only 16% of the variance and are obviously of minor importance.

The following example from biomedical research shows the utility of the present analysis for providing a statistically valid rationale selection of components for principal components analysis. Specifically, empirical data often consist of a high dimensional set of observed variables. For example, we have previously analyzed the sources of variance of pain thresholds to six different nociceptive stimuli, i.e., thermal (heat or cold), electrical or mechanical (blunt or punctate pressure) pain stimuli [3]. Some of these variables showed a (linear) correlation with others. For dimensionality reduction without losing too much information and a conversion of the possibly correlated variables into a set of values of linearly uncorrelated variables, a principal component analysis (PCA) was used. This resulted in eight variance components (Table 2). Setting the limit of the number of principal components, PCs, (Fig 5) at the traditionally advised Kaiser-Guttman criterion of an eigenvalue > 1 of the covariance matrix [11,12] resulted in three major sources of variance that could be used to interpret the most important sources of variance of human pain thresholds. A similar selection of PCs also results when applying the “elbow criterion” in a so-called scree plot of the absolute values of the eigenvalues sorted for decreasing size (Fig 5 top left, red curve). The elbow criterion is estimated as the point where the steep slope to the left of the scree plot levels to a flat slope [13]. The PCs thus identified by these classical criteria as results of this analysis carried high loadings from all pain stimuli (PC #1), from electrical, blunt pressure and thermal pain stimuli (PC #2) or from punctate pressure pain stimuli (PC #3). However, these PCs failed to translate the distinction between thresholds despite the involvement of different receptors in their perception [14].

**Table 2:** Component loadings for a previously reported real-life example of a principal component analysis performed on the intercorrelation matrix among eight pain threshold measurements ([3]; for comparison, see Table 2 in that publication).

Principal component	Explained variance [%]	Eigenvalue	Heat pain threshold	Heat + capsaicin pain threshold	Cold pain threshold	Cold + menthol pain threshold	5-Hz sine-wave electrical pain threshold	Blunt pressure pain threshold	Punctate pressure pain threshold	Punctate pressure + capsaicin pain threshold
**PC1**	48	3.834	0.76	0.58	0.8	0.72	0.59	0.58	0.75	0.73
**PC2**	14	1.142	0.06	0.01	-0.49	-0.58	0.52	0.52	0.13	0.08
**PC3**	13	1.061	-0.1	0.43	0.14	0.23	0.4	0.21	-0.56	-0.53
**PC4**	9	0.740	0.29	0.6	-0.24	-0.19	-0.05	-0.45	0.04	0.03
**PC5**	6	0.491	-0.53	0.32	0.03	-0.03	-0.13	0.13	0.05	0.26
**PC6**	5	0.432	0.2	0.13	0.01	-0.09	-0.45	0.36	0.02	-0.18
**PC7**	3	0.208	-0.11	0.03	0.07	-0.04	0.05	-0.05	0.33	-0.28
**PC8**	1	0.092	-0.01	0	-0.22	0.2	-0.02	0.03	0.05	-0.02

The relevant four principal components (PCs) are given in bold font. Without the present method, only PCs #1 - #3 with eigenvalues > 1 [11,12] could be validly retained. The set of three principal allowed to show that all different pain measures shared an important common source of variance (PC1) pain evoked by cold stimuli, with or without sensitization by topical menthol application, by blunt pressure or by electrical stimuli (5 Hz sine waves) shared a common source of variance (PC2), and a further common source of variance e was shared by pain evoked by heat stimuli, with or without sensitization by topical capsaicin application, or by punctate mechanical pressure. However, with applying the here reported method, PC4 can now be also be retained, which singles out heat pain corresponding to the different pathophysiology underlying heat perception.

The present ABC analysis can provide a better alternative to the rather subjective Kaiser-Guttman or Elbow criteria. Specifically, following calculation of precise limits for the obtained eigenvalues, set A contained the largest eigenvalue while set B contained three further eigenvalues (Fig 5). Thus, when disregarding set C with the four smallest eigenvalues, the present analysis provides support to take four eigenvalues into account, instead of three eigenvalues when applying classical limits. The attritional PC, with an eigenvalue of 0.74 that had to be dropped from the results in the classical analyses, carried loadings from Heat + capsaicin pain threshold (see Table 2 in [3]). This better reflects the different molecular biology involved in the perception of heat pain, mediated via ion channels such as TRPV1 and TRPV4 [15], from the perception of pain evoked by other stimuli. Thus, the present ABC analysis substantially improved the identification of the important few among the variance components of pain thresholds. The curvatures of the ABC curve for the eigenvalues correspond to a multimodal probability density function of the data [16]. Moreover, the modified results were not only biologically more meaningful; they also included a larger part of the information contained in the pain thresholds data set. That is, while the Kaiser-Guttman criterion [11,12], requesting an eigenvalue > 1 for a PC to be considered, explained only 75% of the total variance in the pain thresholds, the ABC analysis resulting in n = 4 PCs provided 84% of the total variance explained, to which set A of n = 1 PCs contributed 48% (Fig 5 bottom left).

## Discussion

In the analysis of multivariate biomedical data the usually peremptory application of traditional statistical limits is sometimes perceived as leading to a loss of information that could have been validly drawn from a data set. Without a theoretical basis, however, crossing classical limits cannot be advised. We therefore suggest a method to identify the “important few” from sets of items that show a clear inequality in their distribution and provide a calculation of precise set limits based on mathematical properties of the distribution of the analyze items. The present method is based on a calculated ABC analysis, replacing the traditional subjective estimations of ABC set limits by algorithmically determined optimal limits. The innovation of the present method consists of using minimization of the effort and of the unrealized yield, together with optimization of the slope of the ABC curve to precisely calculate these limits as a basis for a valid selection criterion for items from a set of data or parameters.

ABC analyses have their roots in economic thinking. That is, the success of a business depends on efficiency in the sense that returns are always regarded with respect to the efforts or costs required to obtain them. Therefore a large application domain of ABC analysis is business administration or material management. However, its application into the biomedical domain relates to effect sizes, which are ubiquitously addressed in this field. For example, the modest predictive value of common genetic variants in human traits can be attributed, despite statistically significant effects, to the mostly small effect sizes conferred by these variants [17]. The present ABC approach directly addresses this issue by selecting the “important few”, i.e., those items that confer the relatively largest effect sizes. In this respect, it completely fits with contemporary statistical data analysis approaches and is meant to be used there as the example of pain threshold variance components emphasizes.

Indeed, when exemplary joining genetics and pain thresholds, common functional variants exert small effect sizes [18] but when combined, they are able to predict particular pain phenotypes at an accuracy of 80% [19]. When applying ABC analysis to that data, the variants that have previously been included in the predictive combined genotype were identified as those lying in ABC set A (details not shown). A further example of the utility of the present method in biomedicine is the applicability of the effort versus gain problem to common medical screening test problems. A common desire of physicians in practice is the availability of short and easily applicable tests. This has led to various efforts to create abbreviated tests from comprehensive test batteries, such as a three-item test for olfactory diagnosis derived from a comprehensive 48-item test [20]. The development of this test is, retrospectively, a candidate for an ABC analysis, which could provide the important few olfactory tests items on a statistically valid level rather than the intuitive selection that had been applied when developing the test.

An advantage of the present method is its applicability to small data sets such as the present example of pain threshold data containing only eight data points (the eight eigenvalues obtained by means of PCA). For small numbers of points the ABC analysis relied on the quadratic spline interpolation of the ABC curve. This interpolation has been established as optimal for generating valid Lorenz curves [7], therefore, via the above-explained relation of ABC curves to Lorenz cures this is also valid for ABC curves. Importantly, data preprocessing, typically consisting of adjustments of the data range and or variance, must take into account that ABC curves are invariant to scaling, i.e., multiplication by some constant but not to location, i.e., the addition/subtraction of a constant to the data. In particular ABC curves are only defined for non-negative data points *x*_*i*_ ≥ 0. So a standardization of the data should be restricted to a mapping of the data to unit variance.

**Fig 6:**
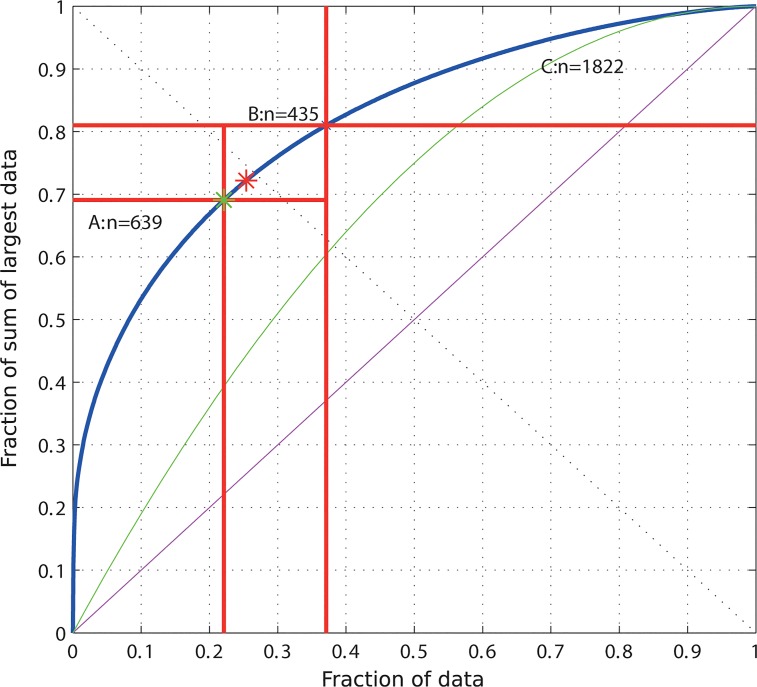
Example analysis of the “SwissInhabitants” data set was taken from an official statistics source. The data consists of the number of inhabitants in the 2896 villages and cities in Switzerland in the year 1900. The analysis shows that 69% of the population lives in 22% of the locations. The ABC curve (blue line) is a plot of *Y*_***i***_, here the cumulative fraction of the Swiss population in 1900, versus *E*_***i***_, here the number of locations (villages or towns in Switzerland). The distribution shows higher inequality than the uniform (green line) and the identity (magenta line) distributions. The red star marks the so called Pareto point *A(A*_***x***_,*A*_***y***_ *)*, i.e., the point at the smallest distance (left oblique black line) to the ideal point at, xy, *E = 0* and *Y = 1*. The green star marks the point on the ABC curve where its slope, *dY/dE*, equals 1. At this point, the profit gain *dABC* equals 1, therefore it is called the *BreakEven* point *B(Bx*,*By)*. The freely available data set on which this analysis was done is also included as an example data set within the above-mentioned “ABCanalysis” R package published on CRAN at http://cran.r-project.org/web/packages/ABCanalysis/index.html.

However, the method is neither restricted to biomedical data nor to small data sets. Another example where it can be applied is taken from demographic analyses. The “SwissInhabitants” data set was obtained from an official statistics source [21] and consists of the number of inhabitants in the 2896 villages and cities in Switzerland in the year 1900. Such data can be explored by the present method to describe the population structure of a country. Applying the present ABC analysis to this data set showed that 69% of the Swiss population lived in only 639 places (22%) in 1900(Fig 6). Nearly 80% of the villages were populated the remaining approximately 30% of the population. A reanalysis of the distribution for the year 1970 shows a concentration effect such that set A now contains only 19% of the cities in which 76% of the Swiss population lives. This somehow resembles the 80/20 rule. The “SwissInhabitants” data set was analyzed in detail in elsewhere [22] and is this freely available data set is also included as an example data set within the above-mentioned “ABCanalysis” R package published on CRAN at http://cran.r-project.org/web/packages/ABCanalysis/index.html.

Working solutions of ABC curve analyses [23] include typical minimum limits for the effort in set A between 0.1 and 0.2, whereas typical maximum limits for the yield in set A are chosen between 0.66 and 0.8 [9,24]. For empirical distributions results of an ABC analysis may usually be consistent with above limits. However, in a $\chi \frac{2}{1}$ distribution, which includes many small values and only a few large values (Fig 3 left), these definitions would hardly allow defining set A since the values of yield in the effort range of 0.1 to 0.2 are below 0.5, hence, requiring precise calculation of these limits. Indeed, scree plots and the elbow criterion are also often used to select an appropriate number of clusters in a cluster analysis [25] and the present method replaces the subjectivity of these approaches.

Finally, the relation of ABC curves to the so-called “Pareto 80/20 Rule”, mentioned above because of its broad recognition, needs clarification. There is no such thing as a “Pareto 80/20-Rule”. Juran has clarified [2] that he mistakenly attributed the 80/20 rule of “roughly 80% of the yield comes from 20% of the effects” to Vilfredo Pareto (1848–1923), who, however, has never published an “80/20-rule”. It should rather be called “Juran 80/20 Rule”. A family of probability distributions, which depend on a parameter α, are called Pareto distributions [26]. For a special value of α = 1.16 the ABC curve of this particular Pareto distribution passes through the point P = (0.2, 0.8). The, so called “80/20 rule” is just the observation that the ABC curve passes in many empirical situations close the point P. If there is a physical law, that systems tend to show ABC curves with the 80/20 rule, it is still unknown [27].

## Conclusions

In this work mathematically defined unique and precise limits for an ABC analysis have been derived. The limits implement the aim of any ABC analysis, i.e., comparing the increase in yield to the required additional effort. In particular, the limit for set A, the “important few”, is optimized in a way that both, the effort and the yield for the other sets (B and C), are minimized. As a typical example from biomedical research, the feasibility of the ABC analysis as an objective replacement for classical subjective limits to select highly relevant variance components of pain thresholds is presented. The method is applicable to many further biomedical problems including the creation of diagnostic complex biomarkers or short screening tests from comprehensive test batteries. Thus, the ABC analysis can be proposed as a mathematically valid replacement for traditional limits to maximize the information obtained from multivariate research data.
